# Identification of Linomide Derivatives as Potential Anticancer Therapeutics using Molecular Docking Studies

**DOI:** 10.3389/fphar.2022.892914

**Published:** 2022-06-16

**Authors:** Giselle A. Borges e Soares, Tanima Bhattacharya, Shivalingrao Mamledesai, Zhaoquan Ai, Alexandru Madalin Hasan, Simona Cavalu

**Affiliations:** ^1^ Department of Medicinal and Biological Chemistry, University of Toledo, Toledo, OH, United States; ^2^ Hubei Collaborative Innovation Centre for Advanced Organic Chemical Materials, Hubei University, Wuhan, China; ^3^ Innovation, Incubation and Industry (i-cube) Laboratory, Techno India NJR Institute of Technology, Udaipur, India; ^4^ Department of Pharmaceutical Chemistry, PES Rajaram and Tarabai Bandekar College of Pharmacy, Goa, India; ^5^ Faculty of Medicine and Pharmacy, University of Oradea, Oradea, Romania

**Keywords:** Mannich reaction, molecular docking, quinolin-2-ones, Linomide, anticancer

## Abstract

12 analogs bearing a structural similarity to Linomide, a bonafide anticancer agent were synthesized wherein cyclization of substituted dianilides rendered 4-hydroxyquinolin-2(1H)-ones that were subjected to a Mannich reaction to yield 4-hydroxy-3-(substituted-1-ylmethyl) quinolin-2(1H)-one analogs. Characterization was performed using IR, ^1^H nuclear magnetic resonance and ^13^C NMR spectral analysis. Subsequently, *in vitro* anticancer studies revealed that Compound 4b showed maximum cytotoxicity with IC_50_ values of 1.539 μM/ml and 1.732 μM/ml against A549 and K562 cell lines respectively. This, however, is lower in comparison with standard Paclitaxel (IC_50_ values of 0.3 μM/ml for both cell lines). Surprisingly, docking studies at the active site of EGFRK revealed Compound 4b possessed a MolDock Score of -110.2253 that is highly comparable to the standard 4-anilinoquinazoline (MolDock Score of -112.04). Our computational and biological data thus provides an insight on the cytotoxicity of these derivatives and warrants future research that can possibly lead to the development of potent anticancer therapeutics.

## Graphical Abstract

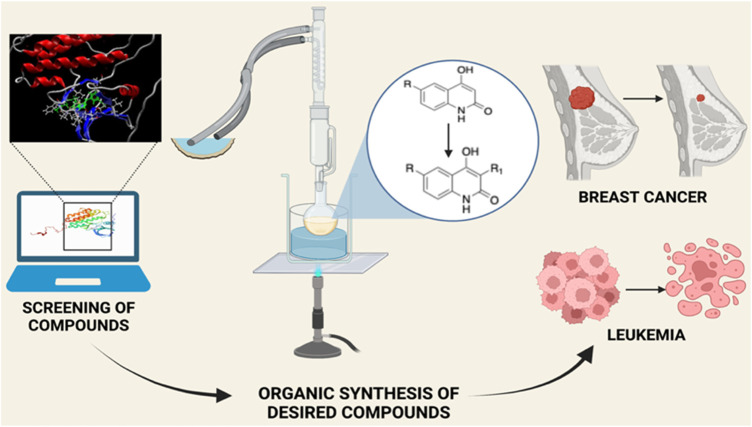

## Introduction

Cancer, a major cause of death world-wide refers to the uncontrollable growth of cells that can metastasize. The localization of cancer at the primary site, is coupled with a good prognosis, however, metastasis, the term used to describe the escape of these cells from the primary site to other distant organs is difficult to treat (Zubair and Ahmad, 2017; Smaranda, 2020) According to the WHO, approx. 10 million lives were lost in 2020 due to cancer, from which lung cancer accounted for 2.21 million cases (WHO, 2021). Due to a better understanding of genetics, cancer pathology and the immune system, several therapies for cancer diagnostics and treatment have been discovered. However, in most instances, treatment failure is observed either due to pharmacological and toxicity issues or drug resistance (Maeda and Khatami, 2018; Islam, et al., 2022).

**FIGURE 1:**
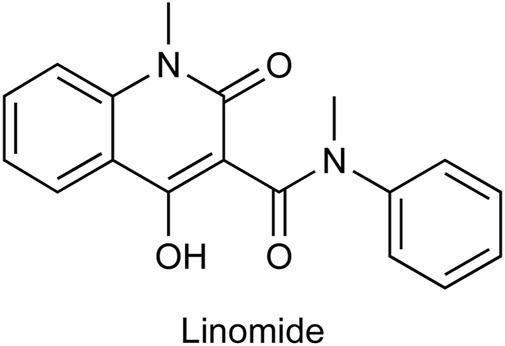
Chemical structure of Linomide, a known anticancer agent and the lead molecule of our current paper.

Linomide, a quinoline-3-carboxamide (Figure 1), an immunomodulatory drug has been shown to possess antibacterial, antiangiogenic, and anticancer activity (Gaude et al., 2017). As the mechanism of action of Linomide is elusive till today, world-wide, researchers have attempted to develop analogs using Linomide as a lead molecule to understand its pharmacological action and biological target. Yang et al. developed Linomide analogs that showed better and a more selective anti-angiogenic activity on endothelial cells as compared to Linomide. These results led the authors to conclude that the selectivity is due to an alternative unknown pathway not involving VEGF/KDR and that requires further investigation (Yang et al., 2005). Ma et al. developed novel histone deacetylase inhibitors for hepatocellular carcinoma using Linomide as a lead molecule. Compounds showed cytotoxicity in Hep G2, and HuH-7 cell lines, the best compound showing an IC_50_ of 1.53 and 3.06 μM respectively (Ma et al., 2021). Another study involved the replacement of the anilido moiety of Linomide at C-3 position with a sulfamoyl acyl, N-methyl, and N-phenyl functionality by Priolkar et al. (2020). These compounds showed superior inhibitory activity through well conserved H-bonding with amino acids at the EGFRK active site observed through docking studies. Moreover, this study indicated that a phenyl ring system at C-3 rendered superior IC_50_ when tested on A549 and K562 cell lines.

In keeping with the above-mentioned study and to further understand the mechanism of action of Linomide, we decided to use Linomide as a “lead-molecule” and to synthesize 12 derivatives bearing a structural resemblance to it through modification at the C-3 position of the quinoline-2-one nucleus using various heterocyclic ring systems. We then performed molecular docking studies which allowed us to conclusively determine the binding site of these analogs thus allowing the prediction and determination of the binding site of Linomide. Following synthesis, we characterized these compounds using Fourier transform infrared spectrometry (FT-IR), ^1^H nuclear magnetic resonance (NMR) and ^13^C NMR spectral analysis in terms of docking results. Finally, to evaluate the anticancer ability of these compounds, we performed the MTT assay.

Hence, by using computational and biological data, the aim of our work was to establish the mechanism by which Linomide behaves as an anticancer drug and warrants future *in vitro* and *in vivo* studies on Linomide derivatives that can be further developed into potent anticancer agents.

## Rationale of Molecular Design

The discovery of the 4-anilinoquinazoline class of EGFRK-targeting anti-cancer drugs was a major step forward in drug discovery as these agents brought about a potent and selective inhibition of tyrosine kinase through competitive binding at the enzyme’s ATP site. The SAR of 4-anilinoquinazoline revealed the requirement of a small lipophilic electron withdrawing group at position three of the aniline ring along with electron donating groups at position six and seven of the quinazoline ring system. Moreover, SAR studies indicated that the -N atom on position one of the quinazoline nucleus is significant for H-bonding formation with Met-769 (Rewcastle et al., 1998). Investigation of the pharmacophoric features shared by EGFRK inhibitors indicated that two features are significant for activity:

1) The presence of a heterocyclic aromatic ring system that takes part in hydrogen bond formation with amino acids in the active site of EGFRK (Ma et al., 2021).
2) A hydrophobic side chain that interacts with the hydrophobic pocket of EGFRK (Rewcastle et al., 1998; Priolkar et al., 2020).

In keeping with this observation, with respect to the synthesis of all Linomide derivatives described in this paper, we decided to replace the -CH_3_ group attached to the -N atom at position one of Linomide with a -H atom to determine its role in -H bonding with amino acids at the EGFRK groove. Moreover, we also decided to modify position C-3 of Linomide and introduce bulky heterocyclic ring systems such as morpholine, piperidine and pyrrolidine to determine if these groups could improve interactions at the active site of EGFRK. Since the presence of an electron-withdrawing group was also found to be necessary for activity, we decided to synthesize analogs bearing groups such as -Cl, -Br, -F at the C-6 position to determine the significance of the C-6 position as well as the role of these functionalities in bringing about an inhibition of EGFRK.

## Results and Discussion

### Chemistry

**SCHEME 1:**
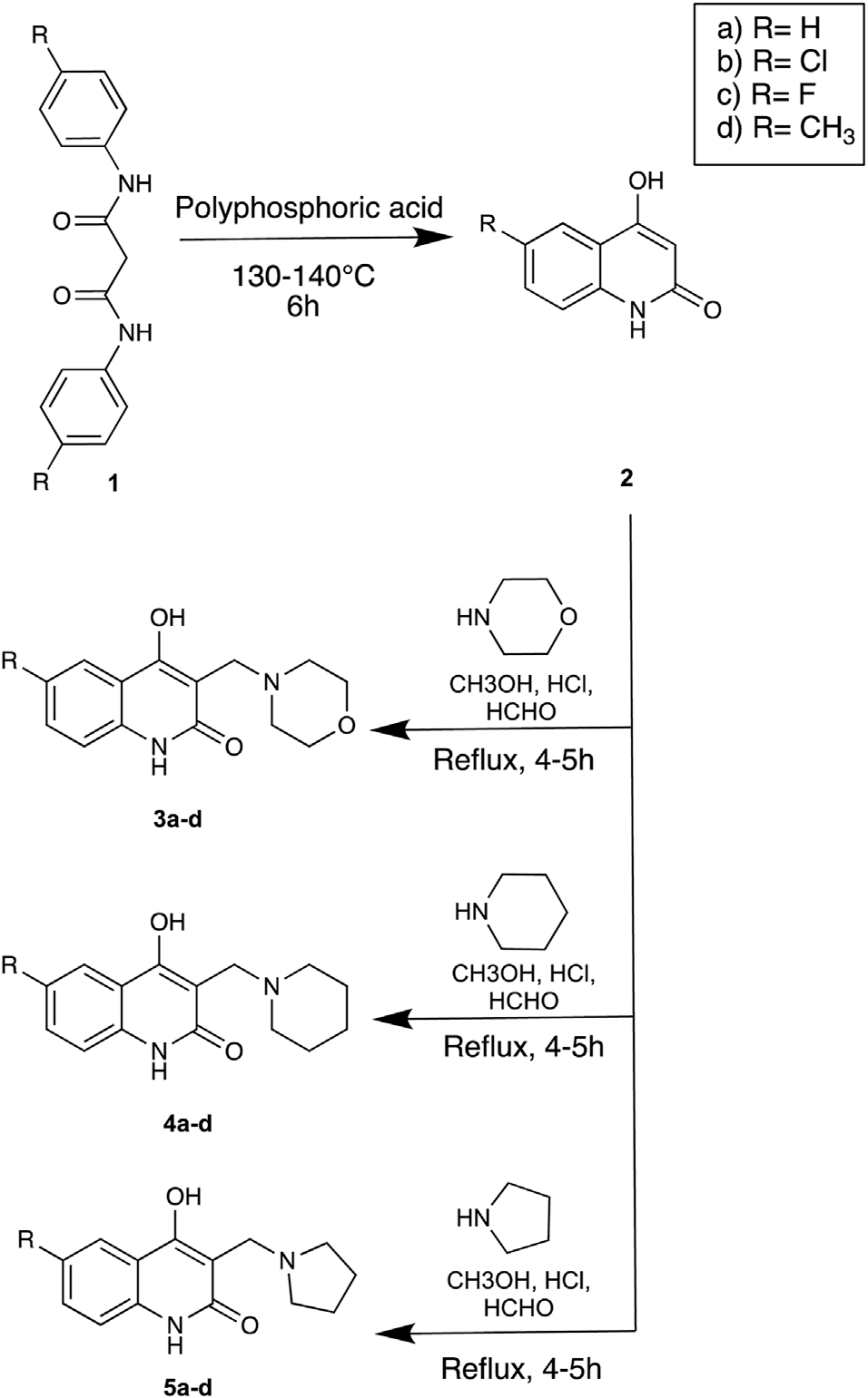
Strategy for the synthesis of Linomide derivatives described in this paper.

The strategy implemented for the synthesis of 4-hydroxy-3-(substituted-1-ylmethyl) quinolin-2(1H)-one derivatives is described in Scheme 1. The starting material constituting of dianilides N1, N3- diphenyl/bis(4-chlorophenyl)/bis (4- fluorophenyl)/di-(p-tolyl) malonamide (1a/1b/1c/1d) were synthesized as described in the literature (Mokrov et al., 2019). Table 1 illustrates the structures of the intermediates and synthesized compounds along with their IUPAC names.

**TABLE 1:** Summary of the reagents, intermediates, synthesized compounds, their labels, IUPAC names and functional groups.

Label	IUPAC name	Structure
1a	2-phenyl-*N*-(phenylcarbamoyl)acetamide	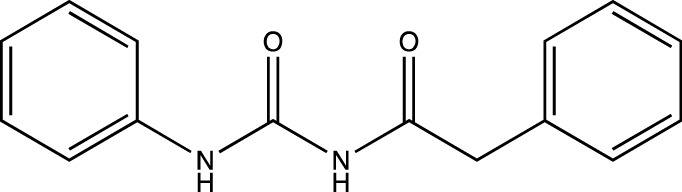
1b	2-(4-chlorophenyl)-*N*-((4-chlorophenyl) carbamoyl)acetamide	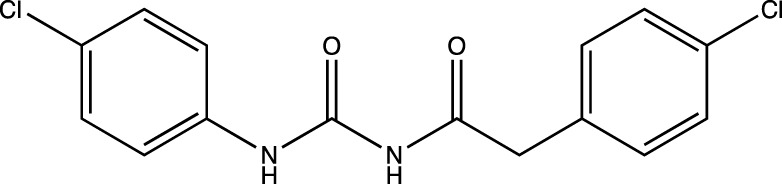
1c	2-(4-fluorophenyl)-N-((4-fluorophenyl) carbamoyl)acetamide	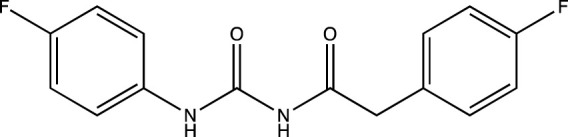
1d	2-(*p*-tolyl)-*N*-(*p*-tolylcarbamoyl)acetamide	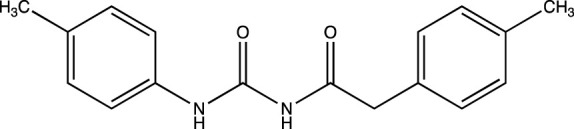
2a	4-Hydroxyquinolin-2(1H)-one	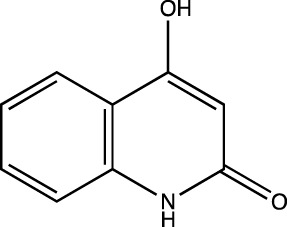
2b	6-chloro-4-hydroxyquinolin-2(1*H*)-one	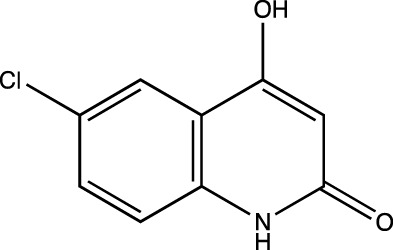
2c	6-fluoro-4-hydroxyquinolin-2(1*H*)-one	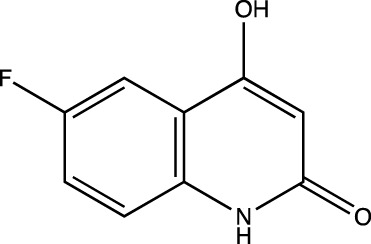
2d	4-hydroxy-6-methylquinolin-2(1*H*)-one	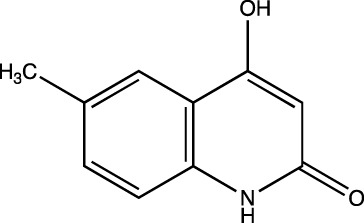
3a	4-hydroxy-3-(morpholinomethyl)quinolin-2(1*H*)-one	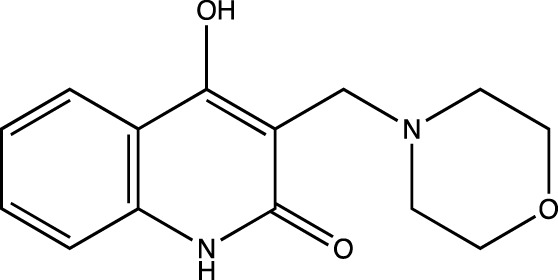
3b	6-chloro-4-hydroxy-3-(morpholinomethyl) quinolin-2(1*H*)-one	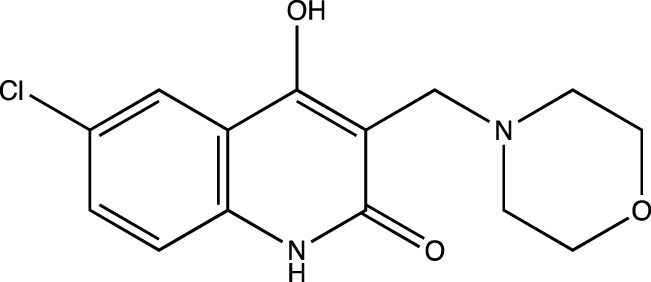
3c	6-fluoro-4-hydroxy-3-(morpholinomethyl)quinolin-2(1*H*)-one	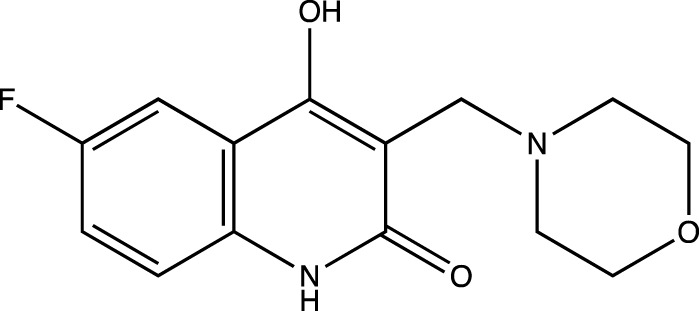
3d	4-hydroxy-6-methyl-3-(morpholinomethyl)quinolin-2(1*H*)-one	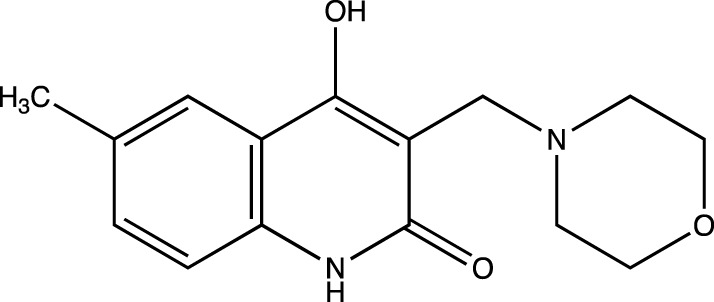
4a	4-hydroxy-3-(piperidin-1-ylmethyl)quinolin-2(1*H*)-one	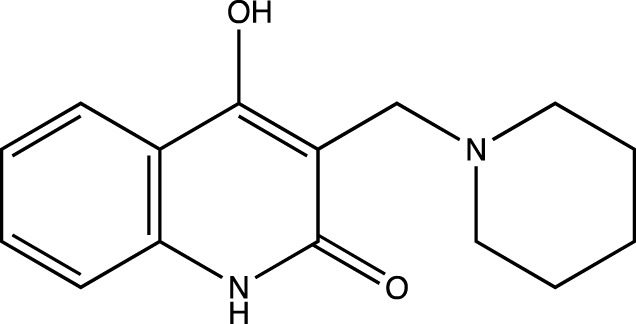
4b	6-chloro-4-hydroxy-3-(piperidin-1-ylmethyl)quinolin-2(1H)-one	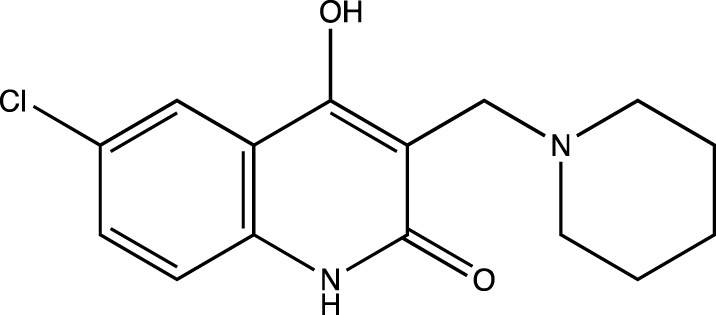
4c	6-fluoro-4-hydroxy-3-(piperidin-1-ylmethyl)quinolin-2(1*H*)-one	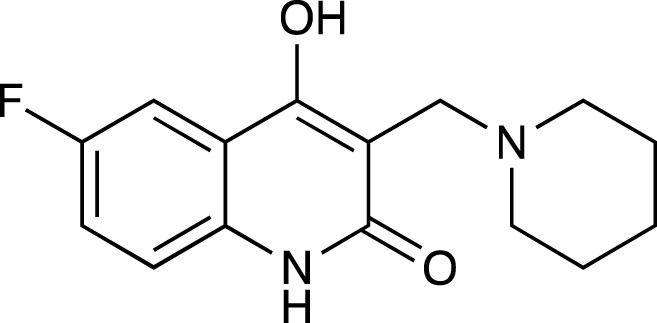
5a	4-hydroxy-3-(pyrrolidin-1-ylmethyl)quinolin-2(1H)-one	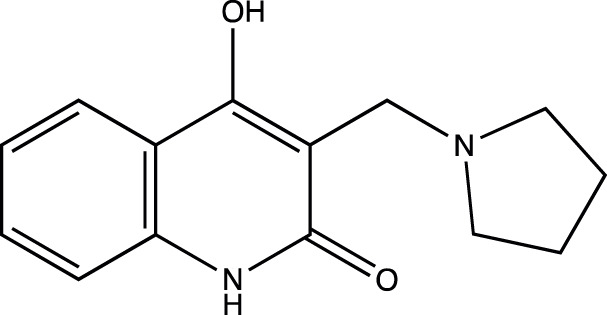
5b	6-chloro-4-hydroxy-3-(pyrrolidin-1-ylmethyl)quinolin-2(1*H*)-one	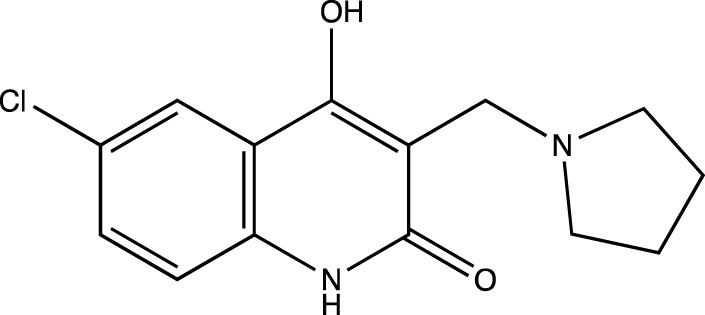
5c	6-fluoro-4-hydroxy-3-(pyrrolidin-1-ylmethyl)quinolin-2(1*H*)-one	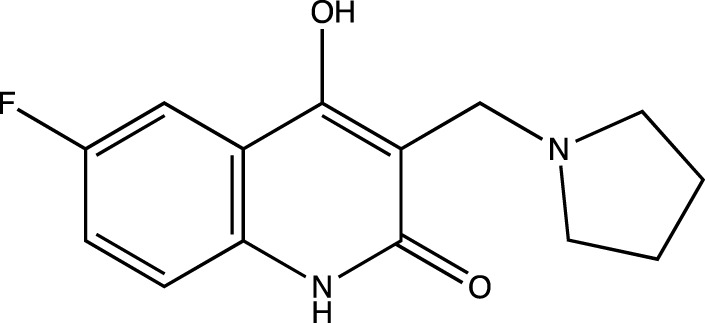
5d	4-hydroxy-6-methyl-3-(pyrrolidin-1-ylmethyl)quinolin-2(1*H*)-one	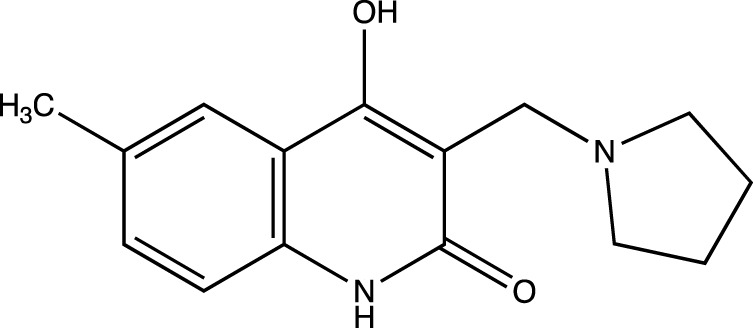

### In Silico Studies

#### Molecular Docking Results

**FIGURE 2:**
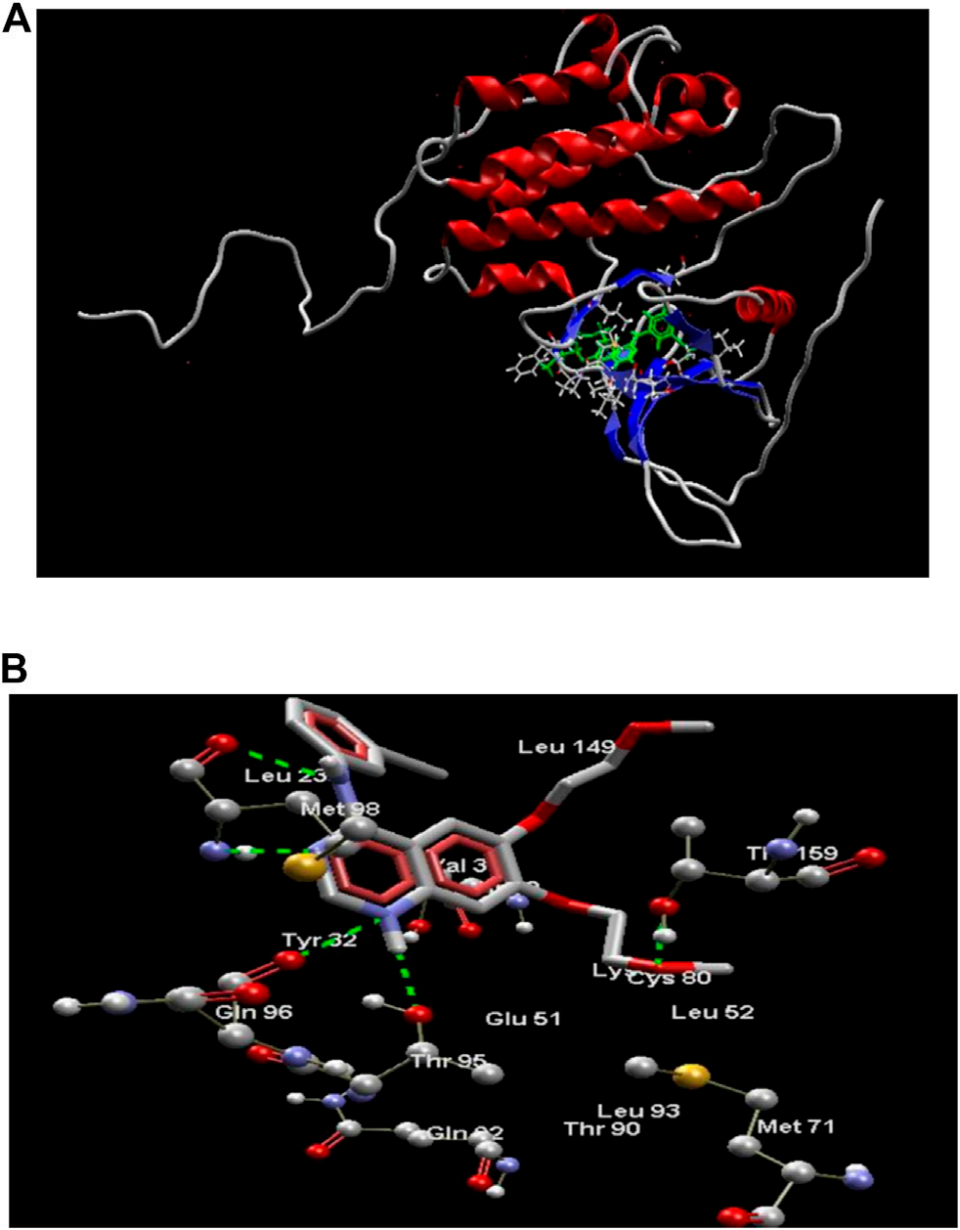
**(A)** Structure of EGFRK domain complexed with 4-AQ (PDB ID: 1m17). **(B)** 4-AQ docked in its best conformation (pose) into the active site of EGFRK.

The results obtained upon performing docking studies using the MoleGro virtual docking software is summarized in Table 2. Docking scores of all 12 synthesized derivatives with the EGFRK enzyme was obtained and were compared to the score obtained by the reference ligand 4-anilinoquinazoline (4-AQ) (a known inhibitor of EGFRK) upon binding to EGFRK as illustrated in Figure 2A and Figure 2B. MolDock scores of the synthesized compounds ranged from -138.886 to -84.6748 while the MolDock score of 4-AQ was -112.04.

**TABLE 2:** MolDock scores of synthesized compounds (3a-d, 4a-d, 5a-d).

Compound	MolDock score	Rerank score	H bond
3a	−138.886	−95.648	−3.98827
3b	−89.8311	−74.2058	−8.1212
3c	−89.8616	−74.6807	−7.59231
3d	−90.4635	−81.2682	−6.38685
4a	−129.335	−100.276	−6.88579
4b	−110.2253	−71.6316	−2.10805
4c	−97.944	−83.0118	−9.02067
4d	−98.5655	−84.6542	−9.42867
5a	−84.6748	−75.7835	−7.5
5b	−88.5125	−77.8308	−7.00347
5c	−88.6646	−77.8062	−6.93734
5d	−88.6957	−78.4212	−7.02415
4-AQ	−112.04	−75.7482	0

As illustrated in Figure 2B, the binding of 4-AQ to EGFK involves four interactions:

1) H-bond formation between -NH at position one of quinazoline and -C=O of Gln96 and -OH of Thr95.
2) H-bond interaction between nitrogen atom at position three of quinazoline and -NH of Met98.
3) H-bond interaction between -NH at position four of quinazoline and -C=O of Met98.
4) Interaction between oxygen from the terminal methoxy group of the side chain with -OH of Thr159.

**FIGURE 3:**
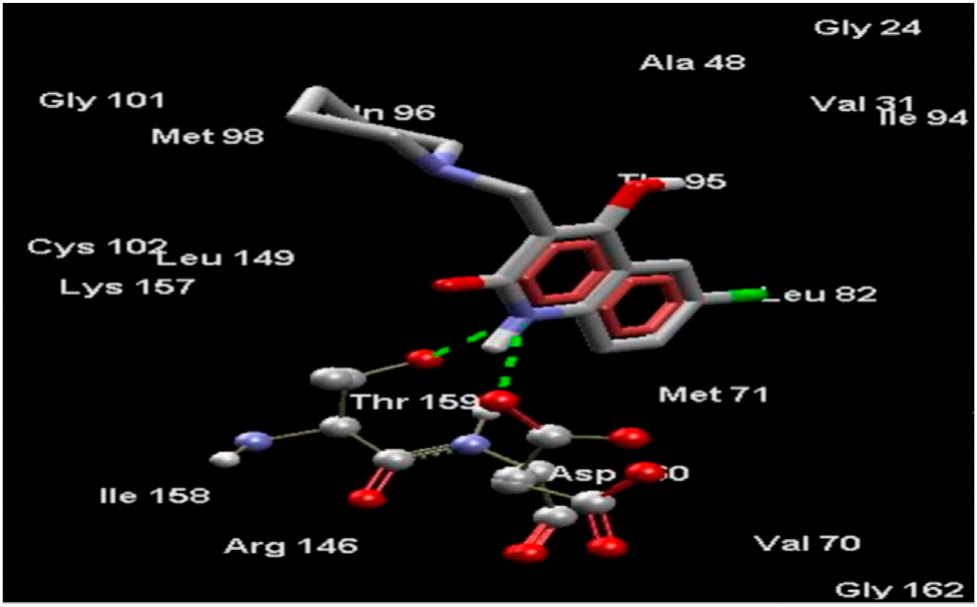
Compound 4b docked in its best conformation (pose) into the binding site of EGFRK.

Amongst all compounds docked, compound 4b had a dock score of -110.2253, a score that was comparable with that obtained with 4-AQ. Upon further analysis, we observed an-NH group interaction of the quinolin-2-one nucleus with both Thr159 and -C=O of Asp160 as illustrated in Figure 3, indicating a good fit. A good fit and a strong non-covalent interaction of the molecule at the EGFRK site indicates its potential to be highly therapeutic and effective as a pharmacological agent.

Based on the molecular docking scores, we performed the MTT assay of representative derivatives that had highest, lowest, and comparable docking scores to that of 4-AQ to determine the correlation between docking results obtained and *in vitro* studies as well as to determine their ability to behave as potential anticancer agents.

### Biological Evaluation

#### MTT Assay

Cell line of A549 (Millipore Sigma, 86012804) cultured in Dulbecco’s Modified Eagle’s Medium (DMEM) (Thermofischer, Cat. No: 11965-092) supplemented with 10% heat inactivated fetal bovine serum (HI-FBS) (Gibco, Invitrogen, Cat. No: 10270106) and cell line of K562 (Millipore Sigma, 89121407) cultured in RPMI (HiMedia Laboratories, Cat. No: AL180A) were supplemented with 1% antibiotic-antimycotic 100X solution (Thermofischer Scientific, Cat. No: 15240062) and then seeded at a density of approximately 5 × 10^3^ cells/well in a 96 well flat bottom microplate and maintained overnight at 37°C ± 5% CO_2_ and 95% humidity. Synthesized compounds dissolved in 1X PBS were then used for treatment in concentrations of 400, 300, 200, 100, 50 and 25 μg/ml and cells were further incubated for 48 h. The cells were washed twice with 1X PBS followed by the addition of 20 μL of the MTT staining solution and incubation at 37 °C. After 4 h, 100 μL of di-methyl sulfoxide (DMSO) was added to dissolve the formazan crystals and the absorbance was recorded at 570 nm using microplate reader. The formula used to determine surviving cells and inhibitory cells was as follows:

$$\mathrm{\% Surviving cells}=\;\frac{\mathrm{Mean Optical density}\left(\mathrm{OD}\right)\mathrm{of test compound}}{\mathrm{Mean OD of negative control}}\;\times \;100$$

% Inhibiting cells=100−Surviving cells

IC_50_ of compounds 3a (MolDock score -138.886), 4a (MolDock score -129.335), 5a (MolDock score -84.6748) and 4b (MolDock score -110.2253) were measured using the MTT Assay which are illustrated in Table 3 for the A549 cell line and Table 4 for the K562 cell line. These compounds were compared with standard Paclitaxel that showed an IC_50_ of 0.3 μM in both A549 and K562 cell lines.

**TABLE 3:** IC_50_ values of synthesized compounds against A549 cell line.

Compound	IC_50_^a^ (μM/ml)
3a	1.632
4a	2.039
5a	2.249
4b	1.539
Paclitaxel	0.3

IC_50_^a^ are the mean±S.D, of 3 separate experiments.

**TABLE 4:** IC_50_ values of synthesized compounds against K562 cell line.

**Compound**	**IC_50_^b^ (μM/ml)**
3a	1.978
4a	2.191
5a	2.808
4b	1.732
Paclitaxel	0.3

IC_50_^b^ are the mean± S.D, of 3 separate experiments.

### SAR of Synthesized Linomide Analogs

Our *in-silico* data as well as the data we obtained through the biological assay performed on A549 and K562 cell lines sheds light on the significance of certain positions of the quinoline-2-one nucleus of Linomide as well as the presence of certain functionalities. Here, in this paper, we report the significance of N-1, C-3, and C-6 of the quinoline-2-one nucleus.

Upon performing docking studies on all the synthesized compounds, it was revealed that the -N atom at position one plays a very important role in H-bonding interactions. As all our analogs possessed a hydrogen atom bonded to N-1 of the quinoline-2-one nucleus, all the compounds synthesized showed two hydrogen-bond interactions with Thr159 and Asp160 present at the active site of EGFRK. This is illustrated in Figure 3 with compound 4b, the most potent of the series that showed a MolDock score of -110.2253 which is comparable to that of the standard 4-AQ with a MolDock score of -112.04.

Presence of bulky heterocyclic ring systems at C-3 seems to improve the efficacy and anticancer activity. We observed that analogs possessing the 6-membered morpholine ring system or the piperidine ring system showed better docking scores that were comparable with the standard while analogs bearing a substitution with a 5-membered ring system such as pyrrolidine at C-3 showed diminished MolDock scores. These scores coupled with our results obtained when the analogs were evaluated using the MTT assay reveal that a 6-membered ring system offers better anticancer activity than a 5-membered system.

To determine the significance of the C-6 position of the quinoline-2-one nucleus, we synthesized analogs bearing electron withdrawing groups and electron donating groups. We observed that a substitution of -H or -Cl at C-6 rendered analogs that showed superior MolDock scores and possessed better anticancer activity. However, substitution with -F or -CH_3_ showed inferior in silico and MTT assay results.

## Methodology

### Chemicals and Apparatus

Reagents and solvents used for experimentation were of laboratory grade (SD Fine- ChemLimited, Mumbai; Molychem, Mumbai, India). Reaction monitoring was per-formed using thin-layer chromatography (TLC) with pre-coated Silica gel-G plates. Purification was performed through recrystallization. Thiele’s melting point apparatus was used to determine the melting points of all synthesized compounds. FT-IR spectra of all derivatives were recorded with Shimadzu IR AFFINITY-1 spectrophotometer using the KBr pellet technique. ^1^H NMR and ^13^C spectra data was obtained on Bruker Advance II 400 NMR Spectrometer using CDCl_3_ or DMSO-d_6_ as a solvent. Chemical shifts are expressed as δ (ppm) values.

### Experimental

#### General Procedure for the Synthesis of 2a-d

The dianilide [1a-d] (1 mol) was placed in a round bottom flask and treated with 10 times (by weight) of polyphosphoric acid. The resultant viscous material was heated at 130–140°C for 6 h, followed by pouring on to crushed ice which was made alkaline with 4N NaOH leading to formation of precipitate which was filtered off. The filtrate was washed with diethyl ether (3 × 5 ml). The aqueous phase was collected and acidified with 6N HCl to pH one in an ice bath, resulting in the precipitation of the substituted four- hydroxyquinolin-2(1H)-one product (2a-d). The product was filtered, washed with water, and dried in a desiccator over P_2_O_5_ for 24 h. This product was used without further purification.

#### General Procedure for the Synthesis of 3a-d, 4a-d, 5a-d

The solution of secondary amine (1 mmol) and formaldehyde (10 mmol) was stirred for 5 min, and a solution of compound [2a-d] (0.01 mmol) in methanol was added and refluxed for 4–5 h. The solution was then kept overnight to precipitate the product, filtered using suction, and recrystallized using ethanol. Upon treatment of 2a-d with secondary amines and formaldehyde (Mannich reaction), Mannich bases 3a-d, 4a-d, 5a-d were formed.

##### 4-Hydroxyquinolin-2(1H)-One (2a)

C_9_H_7_NO_2_. Yield: 31.57%. m. p.: >300°C. FT-IR (KBr, cm^−1^): 3093.82 (-NH), 2953.02 (aromatic, -CH), 2906.73 (aromatic, -CH), 2860.43(aromatic, -CH) and 2821.86 (aromatic, -CH), 1662.64 and 1633.71 (-C=O). ^1^H NMR (DMSO-d_6_, δ ppm): 11.1817 (s, 1H, -OH), 8.1777 (s, 1H, -NH), 7.0975-7.8063 (m, 4H, -Ar), 5.7947 (s, 1H, -CH).

##### 6-Chloro-4-Hydroxyquinolin-2(1H)-One (2b)

C_9_H_6_NO_2_Cl. Yield: 25.61%. m. p.: >300°C. FT-IR (KBr, cm^−1^): 3250.50 (-NH), 3122.75, 3100.50 (-Aromatic–CH), 2933.73 (-CH), 2854.65 (-CH), 1649.14 (-C=O), 1610.56 (-C=O). ^1^H NMR (DMSO-d_6_, δ ppm): 11.8215 (s, 1H, -OH), 11.3188 (s, 1H, -NH), 7.2655-7.7340 (m, 3H, -Ar), 5.8117 (s, 1H, -CH). 13C (DMSO-d6, δ ppm): 163.32 (1C, -C=O), 161.44 (1C, -C-OH), 137.87 (1C, -C=C- of aromatic ring) 130.65 (1C, -C-Cl aromatic ring), 125.06 (1C, -C=CH aromatic ring), 121.72 (1C, -C=CH aromatic ring), 117.08 (1C, -C=C- aromatic ring), 116.36 (1C, -C=CH aromatic ring), 99.11 (1C, -C=C).

##### 6-Fluoro-4-Hydroxyquinolin-2(1H)-One (2c)

C_9_H_6_NO_2_F. Yield: 25.42%. m. p.: >300°C. IR (KBr, cm^−1^): 3088.03 (Aromatic–CH), 2947.23(-CH), 2899.01 (-CH), 1658.78(-C=O), 1604.77 (-C=O). ^1^H NMR (DMSO-d_6_, δ ppm): 11.7305 (s, 1H, -OH), 11.2719 (s, 1H, -NH), 8.2135 (s, 1H, -Ar), 7.2744-7.4805 (m, 2H, -Ar), 5.8015 (s, 1H, -CH). ^13^C (DMSO-d_6_, δ ppm): 163.26 (1C, -C=O), 161.62, 161.60 (1C, -C-OH), 157.88 (1C, -C=C- of aromatic ring), 135.83 (1C, -C-F of aromatic ring), 117.58 (1C, -C=CH of aromatic ring), 114.05 (1C, -C=CH of aromatic ring), 113.00 (1C, -C=C- of aromatic ring), 111.06 (1C, -C=CH of aromatic ring), 99.06 (1C, -C=C).

##### 6-Methyl-4-Hydroxyquinolin-2(1H)-One (2d)

C_10_H_9_NO_2_. Yield: 30.09%. m. p: >300°C. IR (KBr, cm^−1^): 3138.18 (Aromatic–CH), 3024.38 (Aromatic–CH) 2976.16 (-CH), 2920.23 (-CH), 2862.36 (-CH), 1670.35(-C=O), 1608.63 (-C=O).

##### 4-Hydroxy-3-(Morpholinomethyl) Quinolin-2(1H)-One (3a)

C_14_H_16_N_2_O_3_. Yield: 83.10%. m. p.: >300°C. IR (KBr, cm^−1^): 3078.39 (Aromatic–CH), 2943.37 (-Aliphatic–CH), 2872.01 (-Aliphatic–CH), 1656.85 (-C=O), 1606.70 (-C=O). ^1^H NMR (DMSO-d_6_, δ ppm): 12.7076 (s, 1H, -OH), 12.0977 (s, 1H, -NH), 7.2234-7.9370 (m, 4H, -Ar), 3.8284 (s, 2H, -CH_2_), 2.5167- 2.5344 (m, 8H, -morpholine). ^13^C (DMSO-d_6_, δ ppm): 166.52 (1C, -C=O), 160.53 (1C, -C-OH), 136.82 (1C, -C=C-), 130.88 (1C, -CH = CH of aromatic ring), 122.86, 122.84 (1C, -CH = CH of aromatic ring), 115.98, 115.96 (2C, -C-CH, -C=C- of aromatic ring), 109.15 (1C, -C=C- of aromatic ring), 70.04, 70.02 (2C, -CH_2_, morpholine), 62.98, 62.96 (2C, -CH_2_, morpholine), 55.97 (1C, -CH_2_).

##### 6-Chloro-4-Hydroxy-3-(Morpholinomethyl) Quinolin-2(1H)-One (3b)

C_14_H_15_N_2_O_3_Cl. Yield: 86.47%. m. p.: >300°C. IR (KBr, cm^−1^): 3093.82 (Aromatic–CH) 2997.38(Aliphatic–CH), 2956.87(Aliphatic–CH), 2808.36(Aliphatic–CH), 2644.41 (Aliphatic–CH), 1654.92(-C=O)., 1606.70 (-C=O).^1^H NMR (DMSO-d6, δ ppm): 12.6207 (s, 1H, -OH), 12.2501 (s, 1H, -NH), 7.3891-7.8623 (m, 3H, -Ar), 3.2204 (s, 2H, -CH_2_), 2.4984-2.5166 (t, 8H, -morpholine).

##### 6-Fluoro-4-Hydroxy-3-(Morpholinomethyl) Quinolin-2(1H)-One (3c)

C_14_H_15_N_2_O_3_F. Yield: 36.058%. m. p.: >300°C. IR (KBr, cm^−1^): 2954.95(-CH stretch), 2924.09(-CH stretch), 2852.72 (-CH stretch), 1658.78(-C=O), 1612.49 (-C=O).

##### 4-Hydroxy-6-Methyl-3-(Morpholinomethyl) Quinolin-2(1H)-One (3d)

C_15_H_18_N_2_O_3_. Yield: 49.18%. m. p.: >300°C. IR (KBr, cm^−1^): 2954.95(-CH stretch), 2924.09(-CH stretch), 2854.65 (-CH stretch), 1656.85 (-C=O).

##### 4-Hydroxy-3-(Piperidin-1-Ylmethyl) Quinolin-2(1H)-One (4a)

C_15_H_18_N_2_O_2_. Yield: 52.0%. m. p.: >300°C. IR (KBr, cm^−1^): 3076.46 (Aromatic–CH), 2941.44(Aliphatic–CH), 2906.73(Aliphatic–CH), 2872.01 (Aliphatic–CH), 1651.07(-C=O),1606.70 (-C=O). ^1^H NMR (DMSO-d_6_, δ ppm): 12.6865 (s, 1H, -OH), 12.0186 (s, 1H, -NH), 7.2037- 7.9460 (m, 4H, -Ar), 3.8852 (s, 2H, -CH2), 2.5381-2.5552 (m, 8H, CH_2_-piperidine), 2.2280-2.3151 (m, 2H, CH_2_ -piperidine). ^13^C (DMSO-d_6_, δ ppm): 163.26 (1C, -C=O), 159.70 (1C, -C-OH), 135.63 (1C, -C=CH of aromatic ring), 133.03 (1C, -C-CH of aromatic ring), 121.64, 121.61 (2C, -CH = CH of aromatic ring), 114.52, 114.53 (2C, -CH = CH of aromatic ring) 109.93 (1C, C=C), 63.04, 63.01 (2C, -CH_2_, piperidine), 55.0 (1C, -CH_2_), 27.02, 27.00 (2C, -CH_2_ piperidine), 21.61 (1C, -CH_2_ piperidine).

##### 6-Chloro-4-Hydroxy-3-(Piperidin-1-Ylmethyl) Quinolin-2(1H)-One (4b)

C_15_H_17_N_2_O_2_Cl. Yield: 69.19%. m. p.: >300°C. IR (KBr, cm^−1^): 2954.95(-CH stretch), 2924.09(-CH stretch), 2854.65 (-CH stretch) 1658.78(-C=O), 1606.70 (-C=O). ^1^H NMR (DMSO-d_6_, δ ppm): 12.7102 (s, 1H, -OH), 12.0608 (s, 1H, -NH), 8.0805 (s, 1H, -Ar), 7.2822-7.3555 (m, 2H, -Ar), 4.5228 (s, 2H, -CH_2_), 2.8535-2.8880 (m, 4H, CH_2_-piperidine), 2.5424-2.7893 (m, 5H, CH_2_-piperidine), 2.4080-2.5386 (m, 1H, CH_2_-piperidine). ^13^C (DMSO-d_6_, δ ppm): 165.58 (1C, -C=O), 160.04 (1C, -C-OH), 140.17 (1C, -C=CH of aromatic ring), 130.11, 130.19 (2C, -C-CH of aromatic ring), 125.02, 125.15 (2C, -CH = CH of aromatic ring), 115.06 (1C, -CH = CH of aromatic ring) 99.17 (1C, C=C), 74.55, 74.57 (2C, -CH_2_, piperidine), 64.03 (1C, -CH_2_), 26.02, 26.09 (2C, -CH_2_ piperidine), 21.55 (1C, -CH_2_ piperidine).

##### 6-Fluoro-4-Hydroxy-3-(Piperidin-1-Ylmethyl) Quinolin-2(1H)-One (4c)

C_15_H_17_N_2_O_2_F. Yield: 56.438%. m. p.: >300°C. IR (KBr, cm^−1^): 2956.87 (-CH stretch), 2924.09 (-CH stretch), 2854.65 (-CH stretch), 2821.86 (-CH stretch), 2644.41 (-CH stretch), 1658.78(-C=O), 1616.35 (-C=O).

##### 4-Hydroxy-6-Methyl-3-(Piperidin-1-Ylmethyl) Quinolin-2(1H)-One (4d)

C_16_H_20_N_2_O_2_. Yield: 35.07%. m. p.: >300°C. IR (KBr, cm^−1^): 2954.95 (-CH stretch), 2922.16 (-CH stretch), 2854.65 (-CH stretch) 1656.85 (-C=O).

##### 4-Hydroxy-3-(Pyrrolidin-1-Ylmethyl) Quinolin-2(1H)-One (5a)

C_14_H_16_N_2_O_2_. Yield: 70.0%. m. p.: >300°C. IR (KBr, cm^−1^): 3076.46 (Aromatic–CH stretch) 2941.44(-CH stretch), 2872.01(-CH stretch), 2752.42(-CH stretch), 2636.69 (-CH stretch), 1651.07(-C=O stretch), 1606.70 (-C=O stretch).

##### 6-Chloro-4-Hydroxy-3-(Pyrrolidin-1-Ylmethyl) Quinolin-2(1H)-One (5b)

C_14_H_15_N_2_O_2_Cl. Yield: 67.91%. m. p.: >300°C. IR (KBr, cm^−1^): 2954.95(-CH stretch), 2924.09(-CH stretch), 2852.72 (-CH stretch), 1658.78(-C=O stretch), 1606.70 (-C=O stretch).

##### 6-Fluoro-4-Hydroxy-3-(Pyrrolidin-1-Ylmethyl) Quinolin-2(1H)-One (5c)

C_14_H_15_N_2_O_2_F. Yield: 42.198%. m. p.: >300°C. IR (KBr, cm^−1^): 2954.95 (-CH stretch), 2924.09(-CH stretch), 2852.72 (-CH stretch), 1658.78 (-C=O), 1620.21 (-C=O).

##### 4-Hydroxy-6-Methyl-3-(Pyrrolidin-1-Ylmethyl) Quinolin-2(1H)-One (5d)

C_15_H_18_N_2_O_2_. Yield: 30.189%. m. p.: >300°C. IR (KBr, cm^−1^): 2954.95(-CH stretch), 2924.09 (-CH stretch), 2854.65 (-CH stretch), 1658.78 (-C=O stretch), 1606.70 (-C=O stretch).

## Conclusion

Twelve derivatives of Linomide with a modification at C-3 of the quinoline-2-one nucleus were designed, synthesized, and characterized by FT-IR, ^1^H-NMR, and ^13^C-NMR. Anticancer activity of the final compounds was evaluated using the MTT Assay. Docking studies using the EGFRK binding site revealed compound 4b containing a -Cl group at position six of the quinoline-2-one nucleus along with a bulky piperidine group at position three of the quinoline-2-one nucleus, was the most active within the series, with a MolDock score of -110.2253 that was comparable to that of the standard inhibitor 4-AQ with a MolDock score of -112.04. Moreover, it also showed the most cytotoxicity with IC_50_ values of 1.539 μM/ml and 1.732 μM/ml against A549 and K562 cell lines, while all the synthesized compounds were found to be less active than the reference standard paclitaxel (IC_50_ = 0.3 μM/ml). Collectively, as our *in silico* and *in vitro* results agree with each other, we conclude that Linomide and the C-3 modified derivatives reported in this paper interact with the EGFRK binding site in a similar manner as that of 4-AQ. Future *in vivo* studies and investigations will therefore allow for the deeper understanding of the essential pharmacophoric features that are required for inhibiting EGFRK and will thus lead to the development of promising pharmacologically active anticancer therapeutics.

## Data Availability

The original contributions presented in the study are included in the article/supplementary material, further inquiries can be directed to the corresponding authors.
